# Ecology of West Nile virus across four European countries: empirical modelling of the *Culex pipiens* abundance dynamics as a function of weather

**DOI:** 10.1186/s13071-017-2484-y

**Published:** 2017-10-26

**Authors:** Thomas A. Groen, Gregory L’Ambert, Romeo Bellini, Alexandra Chaskopoulou, Dusan Petric, Marija Zgomba, Laurence Marrama, Dominique J. Bicout

**Affiliations:** 1 University of Twente, Faculty of Geo-Information Science and Earth Observation, PO Box 217 7500 AE, Enschede, the Netherlands; 2 EID Mediterranee, 165 Avenue Paul Rimbaud, 34184 Montpellier, France; 3 Centro Agricoltura Ambiente “G. Nicoli”, Via Argini Nord 3351, 40014 Crevalcore, Italy; 4 USDA-ARS, European Biological Control Laboratory, Tsimiski 43, 54623 Thessaloniki, Greece; 5 University of Novi Sad, Faculty of Agriculture, Laboratory for Medical Entomology, Trg D. Obradovica 8, Novi Sad, 21000 Serbia; 6 ECDC, European Centre for Disease Prevention and Control, Tomtebodavagen 11A, 17183 Stockholm, Sweden; 7 Biomathematics and Epidemiology EPSP-TIMC, VetAgro Sup, Veterinary Campus of Lyon, F-69280 Marcy l’Etoile, France; 8 Laue - Langevin Institute, Theory Group, F-38042 Grenoble cedex 9, France

**Keywords:** *Culex pipiens*, Time series, Cross-correlation matrices, SARIMA, West Nile virus, Europe

## Abstract

**Background:**

*Culex pipiens* is the major vector of West Nile virus in Europe, and is causing frequent outbreaks throughout the southern part of the continent. Proper empirical modelling of the population dynamics of this species can help in understanding West Nile virus epidemiology, optimizing vector surveillance and mosquito control efforts. But modelling results may differ from place to place. In this study we look at which type of models and weather variables can be consistently used across different locations.

**Methods:**

Weekly mosquito trap collections from eight functional units located in France, Greece, Italy and Serbia for several years were combined. Additionally, rainfall, relative humidity and temperature were recorded. Correlations between lagged weather conditions and *Cx. pipiens* dynamics were analysed. Also seasonal autoregressive integrated moving-average (SARIMA) models were fitted to describe the temporal dynamics of *Cx. pipiens* and to check whether the weather variables could improve these models.

**Results:**

Correlations were strongest between mean temperatures at short time lags, followed by relative humidity, most likely due to collinearity. Precipitation alone had weak correlations and inconsistent patterns across sites. SARIMA models could also make reasonable predictions, especially when longer time series of *Cx. pipiens* observations are available.

**Conclusions:**

Average temperature was a consistently good predictor across sites. When only short time series (~ < 4 years) of observations are available, average temperature can therefore be used to model *Cx. pipiens* dynamics. When longer time series (~ > 4 years) are available, SARIMAs can provide better statistical descriptions of *Cx. pipiens* dynamics, without the need for further weather variables. This suggests that density dependence is also an important determinant of *Cx. pipiens* dynamics.

**Electronic supplementary material:**

The online version of this article (10.1186/s13071-017-2484-y) contains supplementary material, which is available to authorized users.

## Background

*Culex pipiens* is the major vector for West Nile virus (WNV) in Europe [1, 2]. Because of WNV outbreaks in several places in southern Europe, vector control programmes have been set-up or are under evaluation to control *Cx. pipiens* populations [3]. The efficacy of these programmes in reducing the WNV infection risk remains unclear. A major problem in assessing the efficacy of these control programmes is that the incidence of WNV cases and the population dynamics of *Cx. pipiens* show large variation over regions and years [4]. What can be considered as a high density of *Cx. pipiens* in one region can be considered as a low density in another region. This can be influenced by a number of factors, including landscape characteristics and the weather conditions [5, 6]. It is well known that weather conditions, such as temperature, precipitation and relative humidity affect the population dynamics of mosquito species such as *Cx. pipiens* [4], but landscape uses also have an influence [6]. How landscape and weather factors interact on population dynamics and how this affects the generality of statistical descriptions of *Cx. pipiens* dynamics is, however, somewhat unclear. Nevertheless, standardized statistical descriptions of vector dynamics can be useful to serve as a benchmark against which population reductions resulting from control programmes can be compared. This is, for example, how control efficacy in mathematically designed models is tested [7].

Time series analyses based on integrated autoregressive moving-average models (or ARIMA models [8]) offer a powerful tool to analyse dynamics in time series and to attribute possible covariates (such as treatment effects or meteorological conditions) to the observed dynamics in an empirical way. Previous studies that looked into forecasting the dynamics of virus incidences [9–11] or vector dynamics [12] found promising results when applying ARIMA models. These studies managed to make reasonable forecasts of up to a year [10]. However, such models require long time series of high frequency (e.g. weekly) observations of incidence of a virus or its vector and this is not always available. Therefore, many studies rather look at correlations with other variables that might be able to explain fluctuations in vector dynamics (e.g. [13–15]). Moreover, model studies that look at internal dynamics of vector populations (e.g. [4]), can still be improved by including weather conditions as covariates [4, 12].

All empirical modelling studies of vector populations like *Cx. pipiens* vary from each other, and it is hard to find a general pattern. For example, Trawinski & Mackay [12] found for *Cx. pipiens* and *Cx. restuans* populations in Erie County (New York State, USA), that the most significant predictive weather variables were cooling degree-days base 63 (the absolute difference in Fahrenheit degrees between the average temperature of a week and a base temperature) with a negative coefficient and a ponding index with a positive coefficient both at various time lags. Similarly, Chuang et al. [13] found that *Cx. pipiens* abundance was positively influenced by the preceding minimum temperature in the early season and negatively by precipitation during summer and maximum temperature in July and August in Saginaw County (Michigan, USA). But Lebl et al. [14] found that *Cx. pipiens* in Cook County (Illinois, USA) were positively correlated with daytime length of 4 to 5 weeks prior to the observation and temperature 2 weeks prior to the observation, while they were negatively correlated with wind speed averaged over 3 weeks prior to the observation. So the effect of temperature on *Cx. pipiens* dynamics can be either positive or negative, depending on how temperature was quantified, at which location the study was performed, how the temporal aspect was included and which other variables were taken into consideration. Similarly for European populations of *Cx. pipiens*, temperature, daylight hours and soil moisture were found to be most influential on their dynamics [4] but that the dominant land cover influences these dynamics as well [5]. Therefore, Jiang et al. [4] concluded that “Large rates of change of population abundance remain difficult to predict pointing to gaps in understanding of the mechanisms regulating mosquito dynamics”.

**Fig. 1:**
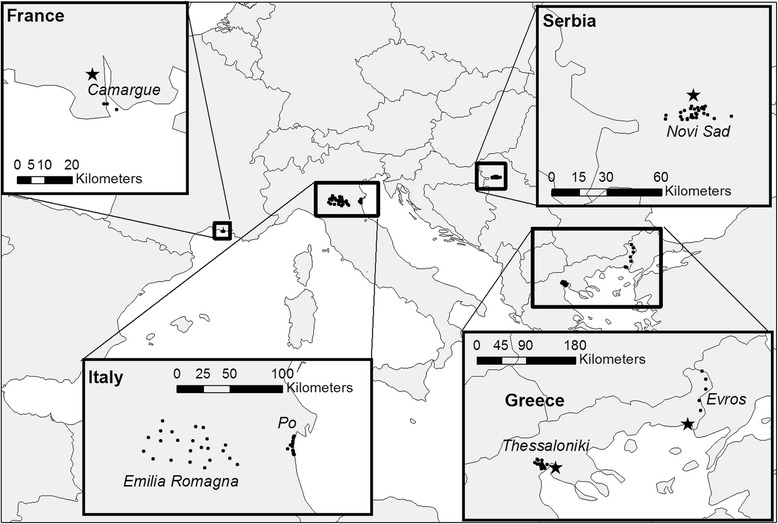
Location of the functional units (FUs) across Europe (see Chaskopoulou et al. [3], for the FU description). Black stars indicate the weather stations and black dots indicate the trap locations within each FU

Therefore, we aim in this study to fit models, based on time series analysis (ARIMA) and on cross-correlation analyses with meteorological data, to describe *Cx. pipiens* dynamics for a number of different sites (Fig. 1) across southern Europe. We tested which types of models perform best to explain these variations, and determined what the commonalities between models fitted for the different sites are. The sites stem from an earlier inventory by Chaskopoulou et al. [3], and we refer to them as functional units (or FUs). These regions vary in climate, size and the way *Cx. pipiens* was monitored.

## Methods

### Data

Data were collected over a number of years for 8 FUs as shown in Table 1 and Fig. 1. An extensive description of the dataset can be found in [3] except for the River Po Delta, for which a description can be found in [16]. For each FU, *Cx. pipiens* density data was collected on a weekly basis, except for Evros and the Camargue, where data was collected bi-weekly (Table 1). For these FUs, data were mapped to weekly by filling gaps in between observations by averaging of those two observations. *Culex pipiens* numbers were collected by several traps per FU. In Greece, CDC light traps baited with CO_2_ were used, while in France, Serbia and Italy traps with CO_2_ only (and no light) were used. In all the cases female mosquitoes were trapped overnight, counted and identified to the species level per trap. To make the dynamics across FUs comparable, *Cx. pipiens* density was expressed as a fraction of the maximum density of *Cx. pipiens* recorded for each trap across the entire time series. Then for each FU, the average relative *Cx. pipiens* density over all traps was calculated. Data on temperature, rainfall, and relative humidity were collected from the weather station nearest to each FU except for the Italian FUs, where data were provided by the regional agency for the environmental protection in Emilia-Romagna region (ARPA-ER) in a gridded format, as listed in Table 1. Data were collected on a daily basis and aggregated to a weekly resolution to match with the mosquito trap data. For the Camargue data on relative humidity were missing, and hence this is not analysed for this FU.

**Table 1:** The functional units (FUs) and the data available per FU

Functional unit	No. of traps	Frequency	Years of data collection	Land cover	Weather data	Weather station
France: Camargue	3	Bi-weekly	2011–2014 (4 years)	Rural (Rice)	Prec, T	Arles, Tour du Valat (43°30′32.04″N, 4°40′03.25″E)
Greece: Evros	8	Bi-weekly	2013–2014 (2 years)	Rural	RH, Prec, T	Alexandroupolis (40°51′09.69″N, 25°57′22.53″E)
Greece: Thessaloniki	14	Weekly	2011–2014 (4 years)	Rural (Rice)	RH, Prec, T	Thessaloniki (40°33′59.04″N, 22°59′23.13″E)
Italy: Emilia-Romagna	25	Weekly	2009–2014 (6 years)	Rural	RH, Prec, T	Daily weather data at 5 × 5 km grid resolution were provided by the ARPA ER-SIM (https://www.arpae.it/sim/)
Italy: River Po Delta	15	Weekly	2005–2014 (10 years)	Coastal		
Serbia: Novi Sad and surroundings	29	Weekly	2000–2007 (8 years)	Mixed urban and rural	RH, Prec, T	Rimski Šančevi (45°19′19.04″N, 19°49′46.75″E)

*Abbreviations*: Prec, precipitation; RH, relative humidity; T, temperature

### Empirical modelling

#### Cross-correlation matrices

**Fig. 2:**
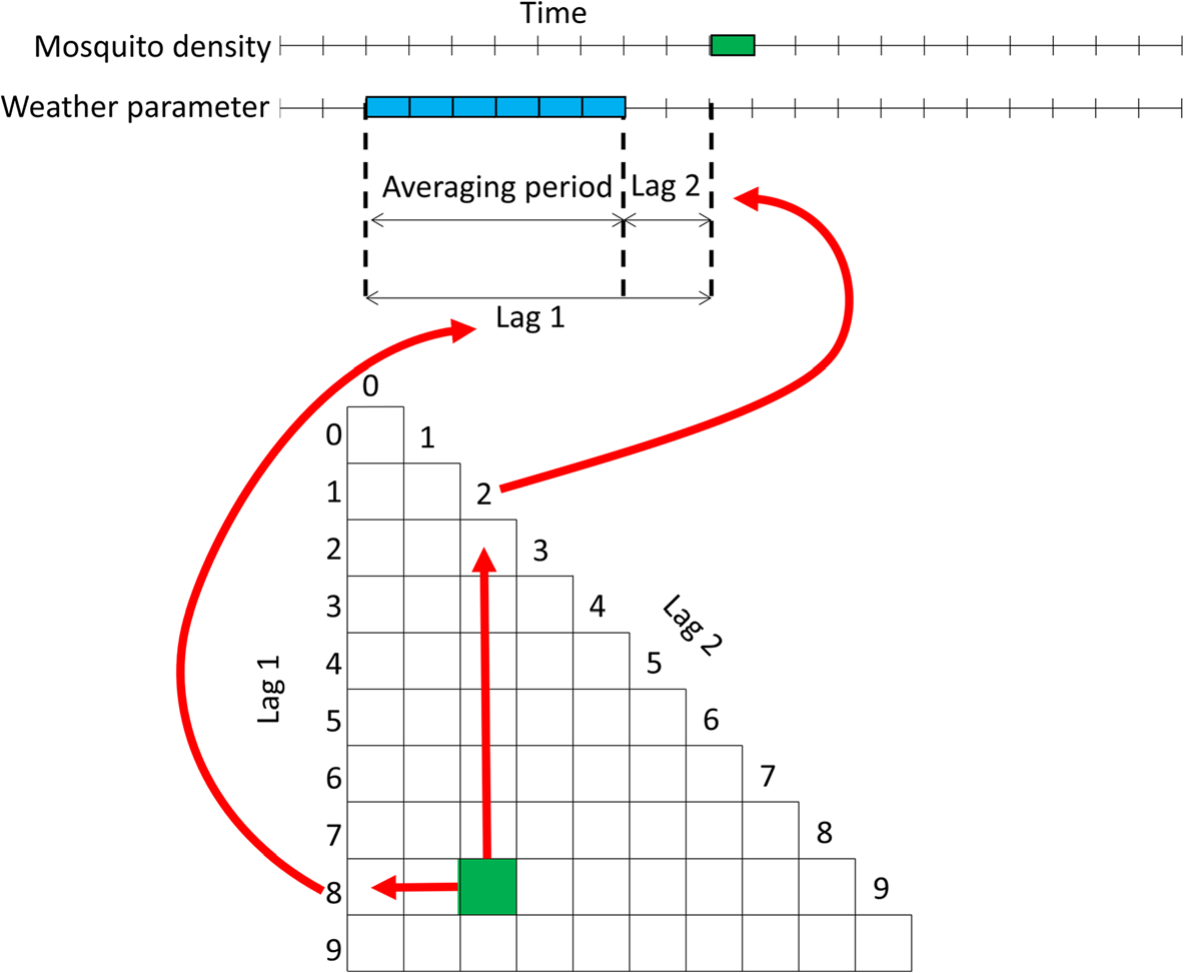
Explanation of the cross-correlation matrices (CCM’s). In cross-correlation matrices, the correlation between the time series of a response variable (mosquito counts from traps in this case) and a time series of the average (or cumulative in the case of precipitation) value for a given period (= lag1 – lag2) at a given lag of an explanatory variable (a weather parameter in this case) is displayed for all possible combinations of periods and lags. Lags are expressed in weeks in line with the used data. When lags are the same, it means only that week is used for calculating a correlation

To find out how correlations between weather conditions and *Cx. pipiens* dynamics vary over time-lags, cross-correlation matrices (CCMs) can provide insights [14]. CCMs give the correlation between the average weather conditions for a given period at a given lag and the number of *Cx. pipiens*. CCMs can be displayed as maps, which indicate the correlations with colours, and the positions (lag1, lag2) in the matrix indicate lag and period (= lag1 – lag2) considered (Fig. 2). We fitted CCMs for all FUs based on average temperature, relative humidity (except for the Camargue) and on cumulative rainfall for the considered period, with lags expressed in weeks. In addition, we fitted a CCM on the interaction between temperature and cumulated precipitation as such interactions have been found relevant for explaining the cases of vector-borne diseases like dengue [17]. All CCMs were generated in R [18] using the *corrplot* package (version 0.77).

#### ARIMA models

Autoregressive integrated moving-average (ARIMA [8]) models estimate the value of an observation (relative *Cx. pipiens* density in this case) at a given time step as a function of earlier observations (i.e. autoregression), the random error of previous observations (the moving average) and a given level of integration (differencing between consecutive time steps). ARIMA models have an order that indicates how many previous time steps are included in the autoregression (p) and the moving average (q). The autoregression order (p) indicates how many previous moments of mosquito density measurements are needed to make a prediction of the next mosquito density. The moving average order (q) indicates how many past deviations from the mean influence the predicted mosquito density at the next time step. Also the integration term comes with an order (d), where 0 stands for no differencing, 1 for a first order differencing etc. The order of an ARIMA model is normally indicated between brackets as (p, d, q). Apart from the effect of previous time steps, the effect of a previous season can be included in what is called a seasonal ARIMA (or SARIMA). In a SARIMA the effects of observations at the same time in the previous year (or years) are taken into account. SARIMA models also have an order indicated by (P, D, Q) analogously to ARIMA models. In our analyses we did not specify the order of our (S)ARIMA models *a priory*, but had an optimal search algorithm (search.arima) as implemented in the *forecast* package [19] in R [18] to make an optimal selection. An optimal selection was defined by finding a model where the AIC (Akaike’s information criterion; see section on model comparison below) was lowest.

After fitting an ARIMA model on the mosquito time series of each FU, these models were extended by including information on weather conditions as covariates into the model. Because in these models there can be also a time lag in the impact of weather conditions on mosquito densities, we fitted CCMs using the residuals of the ARIMA model predictions as a response variable and the weather conditions as explanatory variables. We included the weather conditions in the ARIMA models, averaged over a period and lag that provided the highest correlation according to these CCMs.

#### Model comparison

How these different models compared to each other was tested by looking at improvements in the AIC [8] and the mean absolute error (MAE [19]). AIC is used often to test improvements in fit of a model, as it provides a method to correct possible overfitting of models. Lower values of AIC in absolute sense indicate better fitting models [20]. A difficulty is that AIC cannot be compared for models fitted on different datasets. Therefore AIC was only used to compare goodness-of-fit of models fitted on the same datasets (i.e. the different models fitted per FU). To compare between models fitted on different datasets (so compare across FUs) MAE was used. MAE is a common goodness-of-fit indicator for ARIMA models, when the response variable has been standardized as in our case. An advantage is that MAE is immediately comparable with the original input values, i.e. relative *Cx. pipiens* density. The MAE might be artificially low given the many zero counts outside the *Cx. pipiens* season. Therefore MAE calculations were based only on model predictions in the *Cx. pipiens* season. That season is defined as the period between the first and last “non-zero” observations of *Cx. pipiens* each year.

## Results and discussion

**Fig. 3:**
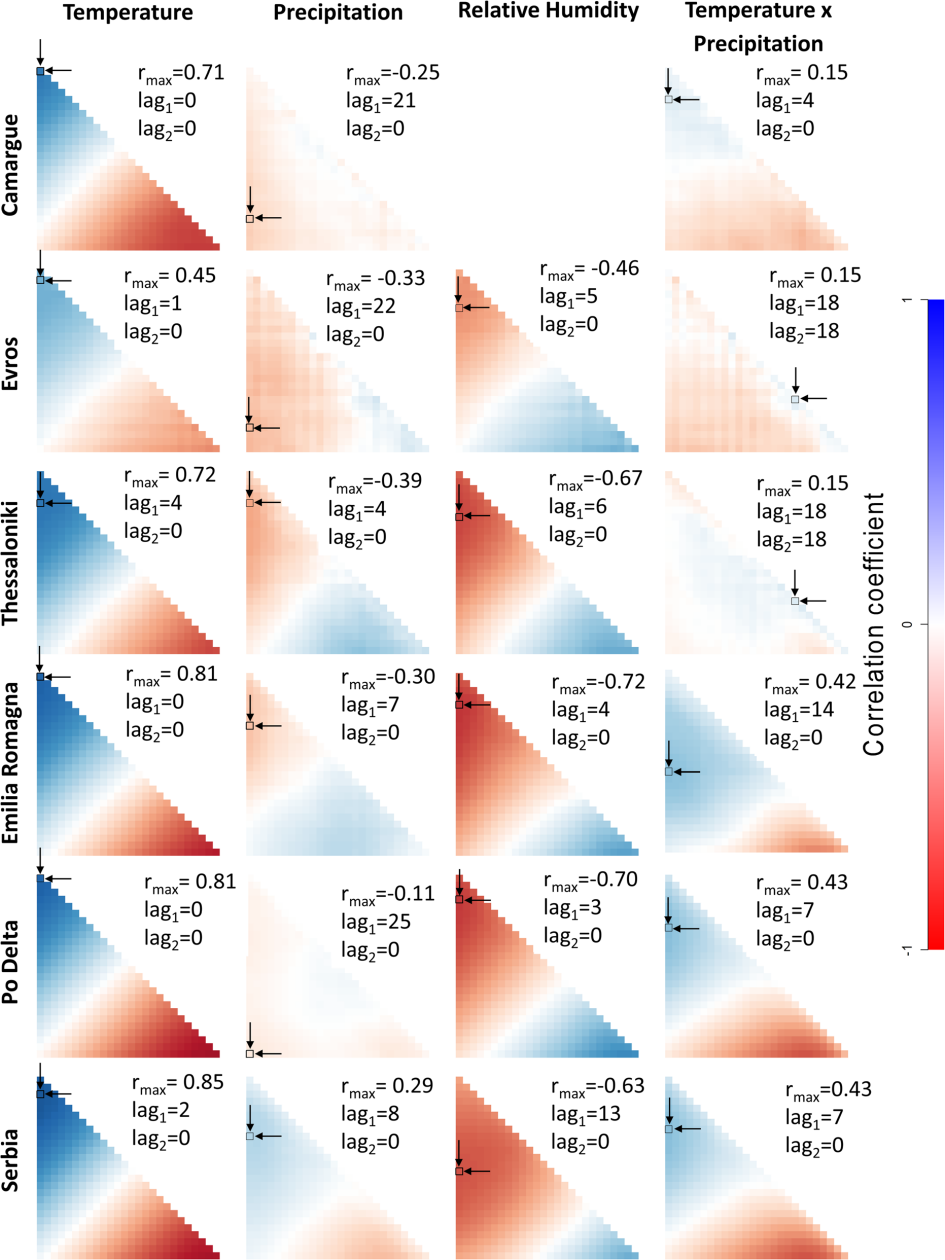
Maps of cross-correlation matrices (CCMs) between the number of *Cx. pipiens* and weather conditions (average temperature, relative humidity and cumulative rainfall). The square (pointed by arrows) on CCMs indicates the maximum correlation as quoted

Maximum (absolute) correlations between lagged weather conditions and relative adult mosquito densities ranged between 0.11–0.85 (Fig. 3). Correlations were fairly strong for temperature and relative humidity, but less strong for precipitation. Also there is a clear gradient across the tested time lags and averaging periods for these two weather conditions, where correlations at longer lags are opposite to correlations at shorter lags. This is not so much the case for precipitation. Because negative correlations can indicate equally relevant relationships in data as positive correlations, we initially determined the strongest correlation as the absolute correlation maximum. Given the gradient for temperature and relative humidity, sometimes the correlation at a very large lag was slightly stronger than the oppositely signed correlation at a shorter lag. For example, the strongest correlation between relative mosquito density and relative humidity in Evros was 0.48 at a lag1 = lag2 = 25 weeks (i.e. the relative humidity of 25 weeks ago only was supposed to have an effect). But at a lag1 of 5 weeks and lag2 of 0 there was a correlation of -0.46. Therefore, we selected the highest correlation (either the most negative or the most positive) that had the shortest lag distance. This also yielded the most consistent results, in the sense that only positive and negative correlations were found with temperature and relative humidity respectively, across FUs (Fig. 3).

The positive correlations with temperature and simultaneously negative correlations with relative humidity can be partly attributed to the negative correlations between temperature and relative humidity (between -0.63 and -0.77, see Additional file 1: Figure S1). Due to this collinearity it is difficult to say which variable is the main determinant of *Cx. pipiens* dynamics. Nevertheless, the maximum correlations with temperature were higher than those for relative humidity. These maximum correlations were also at relatively short lags of up to 4 weeks, but with the majority having no lag (i.e. lag1 = 0 and lag2 = 0; meaning only temperature at the time of the observation has an effect), while those for relative humidity were often longer. This suggests that temperature has a direct positive effect on the number of mosquitoes that are trapped, and that this effect is systematic across FUs. We cannot exclude here a possible bias of temperature on the performance of the traps, rather than that it represents the size of the mosquito population. Nevertheless, we do recognize that high temperatures stimulate the flight activity of *Cx. pipiens* which therefore may enhance the probability to be trapped.

Precipitation showed much less consistent results, and generally weaker correlations. The highest absolute correlation was -0.39, but also positive correlations were found (Serbia). For these maximum correlations, lag1 was generally long (7 up to 25 weeks), while lag2 was consistently 0. A lag2 of 0 means that the cumulative precipitation from lag1 up to the moment considered is calculated. This is suggesting that accumulating precipitation over longer periods before mosquitoes are observed is a better predictor for their dynamics. This might make sense, given the, sometimes erratic, nature of precipitation.

The interaction between temperature and precipitation was generally positive and weak to moderate in correlation with mosquito dynamics; the highest absolute correlation was 0.67.

**Fig. 4:**
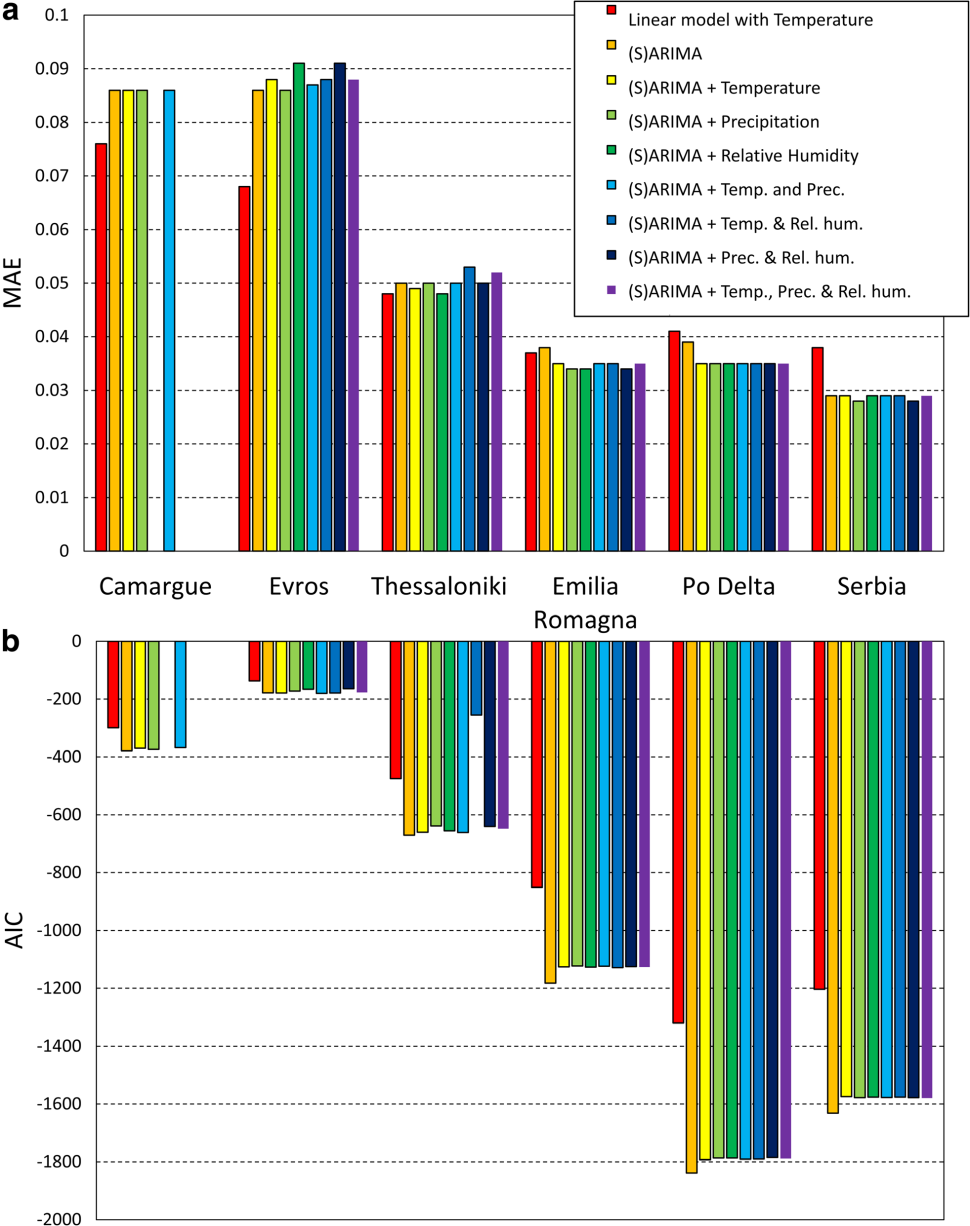
Accuracy indicators. Mean absolute error (MAE) (**a**) and Akaike information criterion (AIC) (**b**) values for the different models fitted to the data, and grouped per functional unit (FU). Values can be found in Additional file 1: Tables S1 and S2

**Fig. 5:**
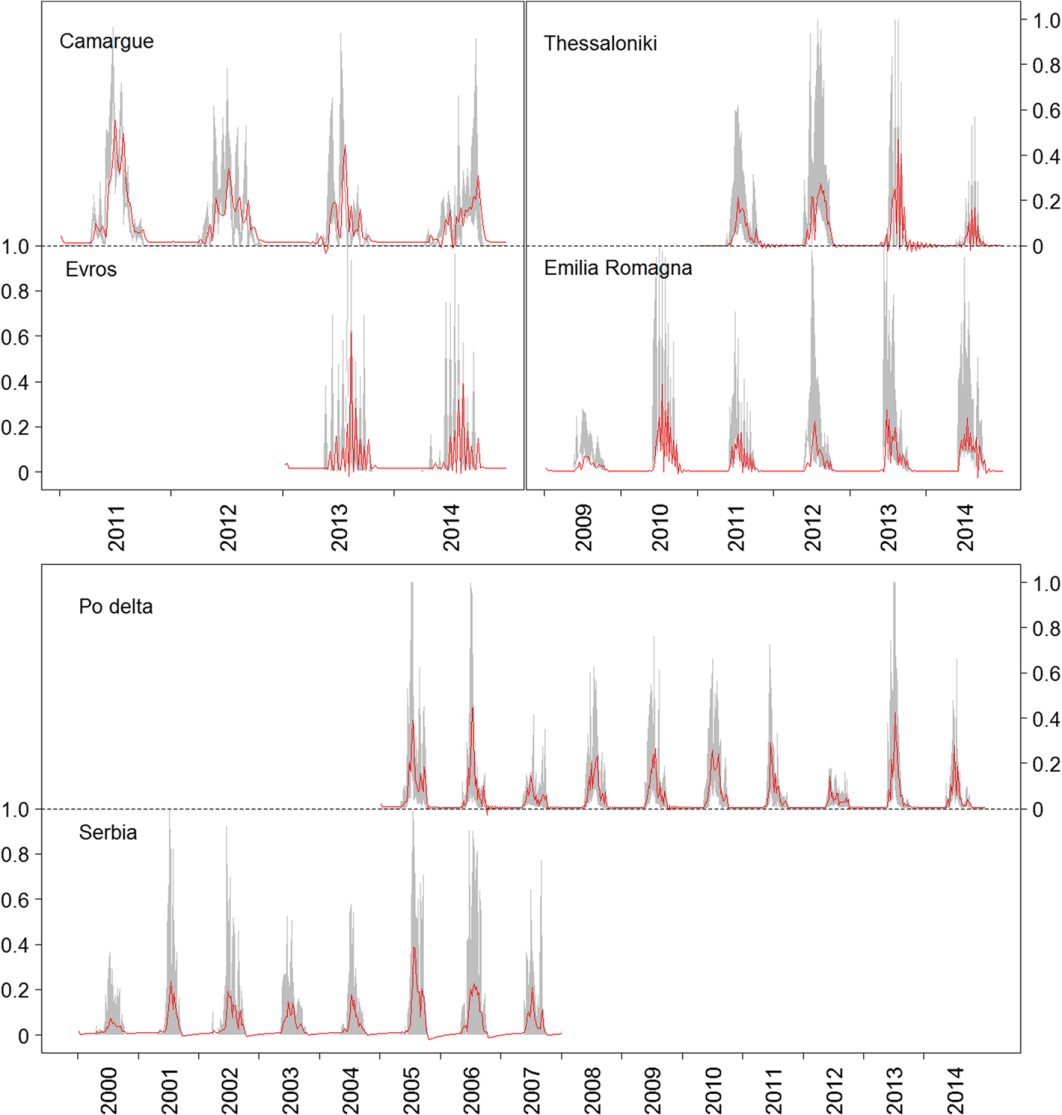
Relative *Cx. pipiens* densities (y-axis) over time (x-axis) and modelled trends. Grey area indicates the 5–95% quantiles of density of all traps in the functional unit, red lines indicate ARIMA predictions

The ARIMA models fitted to the time series data (Table 2) had the smallest error values (MAE) for FUs that had relatively long time series (roughly > 4 years of data) (Fig. 4a) such as the time series for the River Po Delta, Emilia-Romagna and Serbia. For really short time series (< 4 years), like Evros, the error could reach up to almost 10%. To compare the goodness-of-fit for models of one FU, AIC can be better compared than MAE, because it corrects for the number of parameters included in a model. Lower AIC values indicate a better fit. For all FUs, the simple (S)ARIMA, without addition of weather conditions provided the best explanatory power for the dynamics of *Cx. pipiens* (Fig. 4b). This was even the case in the FUs with really short time series, such as Evros and the Camargue. However, the difference in AIC between (S)ARIMA models and linear models was much smaller than for the FUs with longer time series. This can also be seen in Fig. 5, where the match between fitted and observed is much less evident for short time series than for the FUs where longer time series were available. That suggests that when only short time series of mosquito observations are available, modelling the dynamics based solely on climatic conditions can be nearly as good as fitting the more complex (S)ARIMA models, but that when longer time series are available, fitting an (S)ARIMA becomes more advantageous. The longer time series in Fig. 5 shows less erratic fluctuations during the mosquito season and also the timing of peak relative densities and their amplitude is better matched for longer time series than for short time series. The most promising weather condition to model *Cx. pipiens* dynamics directly (by means of a linear model) seems to be temperature at fairly short time lags. Precipitation showed a very inconsistent pattern across the FUs considered in this study. Relative humidity does seem to have a consistent effect on *Cx. pipiens* dynamics as well, although less strong than temperature and of opposite sign (i.e. a negative correlation at short time lags). Given the negative correlation between temperature and relative humidity, part of this explained variation in *Cx. pipiens* dynamics by relative humidity, might be actually explained by temperature, or vice versa. This is a clear case of collinearity, and given the observational nature of this study (as opposed to experimental), it is impossible to separate these two effects totally.

**Table 2:** Fitted (S)ARIMA models with associated order (p, d, q) (P, D, Q) and coefficients

Functional unit (FU)	p		q		P		Q		Intercept
	Order	Coefficient	Order	Coefficient	Order	Coefficient	Order	Coefficient	
France: Camargue	1	0.875	1	-0.316	1	−0.311	1	0.080	0.068
Greece: Evros	1	-0.040							0.044
	2	0.650							
Greece:Thessaloniki	1	-0.872	1	1.096			1	0.212	
	2	0.264	2	0.840					
	3	0.794							
	4	0.407							
Italy: Emilia-Romagna	1	0.0095	1	0.317			1	0.162	0.031
	2	0.759	2	0.164					
			3	-0.102					
Italy: River Po Delta	1	-0.074	1	0.911	1	0.340			0.037
	2	0.671							
Serbia: Novi Sad and surroundings	1	1.682	1	-0.936					0.035
	2	-0.536							
	3	-0.169							

None of the models included a “d” or “D” term. For each FU, the maximum values of p and q correspond to the order “n” of fitted AR (autoregressive) and MA (moving average) models, respectively, and likewise for P and Qin the seasonal models. A model of order “n” is described with n coefficients

Jian et al. [4] investigated to what extent *Cx. pipiens* dynamics was determined by internal dynamics, and to what extent external factors could explain variations in these dynamics. They concluded that *Cx. pipiens* abundance has a significant density dependence at the scale of one week. This matches with our observations. We noticed that all (S)ARIMAS were based on short lag models (in terms of p, d, q, order, see Table 2) and not all models included seasonal effects (P, D, Q order). Partly, this short lag-correlation might have been introduced by the gap-filling for FUs where only bi-weekly data were available (Camargue and Evros). But we also found short lags for other FUs suggesting that the effect of the gap filling is not substantial, compared to the autocorrelation already present in the data. This is, conversely, suggesting that there are density dependent effects. Similarly, Mulatti et al. [21] concluded that including density dependence in combination with key parameters will improve model predictions of mosquito dynamics. As pointed out by Marini et al. [22] such key parameters could be temperatures and precipitation. Nevertheless, Marini et al. [22] also stressed the importance of density dependence in making accurate predictions. We have to note though that long time series of observations (as was also used in [4]*.*) are needed to create models that can make a good prediction based on density dependence.

Jian et al. [4] also found that apart from weather conditions, landscape characteristics (distance to rice fields and satellite based greenness indicators) have an impact on the absolute number of *Cx. pipiens* that can be expected. Because we standardized the dynamics for all the FUs, we cannot make similar inferences. But Carrieri et al. [5] showed that landscape characteristics can also have an effect on the type of dynamics, because in rural areas in the River Po Delta they found a bimodal pattern in seasonal *Cx. pipiens* dynamics, while in urban areas, there was a unimodal pattern. This bimodal pattern was not apparent in the data of our FUs (Fig. 5, see also [3]), but it should be noted that these were aggregated time series across stations. The most likely case would be the Serbian case, where traps were distributed across the city of Novi Sad, but also in the outer regions around the city. In a separate analysis (results not shown) no differences were found in patterns for traps located in the city centre and traps located in the rural periphery of the city.

The aggregation of station data across a spatial unit (referred to as FU in our study) is an approach that averages out fluctuations between stations and gives a more holistic view of mosquito dynamics over an entire area. By standardizing the dynamics relative to the maximum density of mosquitoes trapped, the dynamics have become relative and better comparable across FUs. But at the same time, models fitted to such data have lost their ability to provide absolute predictions of mosquito abundance. But that was also not the main aim of this study. We tried to find commonalities in modelling approaches that would work across FUs. Many studies have made empirical models of *Cx. pipiens* dynamics (e.g. [4, 12, 14, 21–23]), but each study was focussed on one single FU and one particular modelling approach. In this study we tried to identify how well methods applied across different sites. We had variability in site characteristics, including the spatial extent of sites (Fig. 1) number of traps per site (Table 1) and duration of recordings. This allowed us to make inferences about the effect of these site characteristics on modelling *Cx. pipiens* dynamics.

Previous studies on *Cx. pipiens* dynamics looked at many more factors than weather variables alone. For example, land cover variables [4, 5], soil related variables [12], and daytime length [14] have also been identified to explain partly the *Cx. pipien*s dynamics. In this study, we tried to find out how consistent the explanatory power of various conditions would be, and for the presented variables (temperature, relative humidity and rainfall) such data were available across all FUs. Also, these variables can be derived from more generic observation platforms, such as satellites. As was demonstrated in earlier studies [4, 11], satellite-based indicators can play a role in explaining the dynamics of vector populations. This could also be the case for satellite-based weather parameters that can be collected in a standardized way across regions.

However, there are also land use differences between our FUs. The Camargue in France, and the areas around Thessaloniki in Greece are dominantly rural with large extents of flooded rice fields, while the River Po Delta is mainly coastal and the Serbian area around Novi Sad is mainly urban (although traps were scattered across the city as well as the outer areas that are more rural). Precipitation seems to have a negative effect in the more rural areas, but a positive effect in more urban areas (Novi Sad, Serbia), and really very low effects in coastal areas (the Po Delta, Italy). These findings do not allow simply ruling out a relationship between precipitation (or the temperature-precipitation interaction) and mosquito dynamics in urban versus rural areas. Indeed, it is usually considered that containers or artificial habitats where water is stored are independent or weakly dependent on rainfall while ground-fed wetland habitats are more governed by rainfall over the previous few weeks, rather than the instant impact of precipitation. The apparent absence of a clear pattern between precipitation and mosquito dynamics across FUs may be indicative of the heterogeneity of the studied FUs in terms of habitats content and artificial vs ground-fed wetland, regardless their urban or rural land use.

## Conclusions

This study showed that temperature is the most consistent weather variable to predict *Cx. pipiens* dynamics in southern Europe, directly with simple linear models. To a lesser extent relative humidity explains variation as well, although this partly overlaps with temperature due to collinearity. Furthermore, we show that when long time series are available, (S)ARIMA models could be very useful. They can make slightly better predictions of *Cx. pipiens* dynamics, compared to simple linear models based on lagged weather variables. When dynamics are modelled by (S)ARIMAS, lagged weather conditions hardly make any further contribution. This is suggesting that weather dynamics is only relevant to predict *Cx. pipiens* adult dynamics directly, but not to improve more complex models based on time series analysis. The relatively good performance of the (S)ARIMAS also suggests that density dependent mechanisms form an important part of the population dynamics of *Cx. pipiens*.

## Additional file

Additional file 1: AIC (**Table S1**) and MAE (**Table S2**) scores for the different models and correlations between weather variables per functional unit (**Fig. S1**). (PDF 478 kb)

## Abbreviations

AIC: Akaike Information Criterion
CCM: Cross-correlation matrix
FU: functional unit
MAE: mean absolute error
SARIMA: Seasonal Autoregressive Integrated Moving Average method
WNV: West Nile virus
