# Supplementary material for: Ecology of West Nile virus across four European countries: empirical modelling of the Culex pipiens abundance dynamics as a function of weather
Source: Parasit Vectors. 2017 Oct 26;10:524. doi: 10.1186/s13071-017-2484-y (PMC5657042; doi:10.1186/s13071-017-2484-y)

Additional file 1

Ecology of West Nile virus across four European countries: empirical modelling of the *Culex pipiens* abundance dynamics as a function of weather

---

Thomas A. Groen[1], Gregory L'Ambert[2], Romeo Bellini[3], Alexandra Chaskopoulou[4], Dusan Petric[5], Marija Zgomba[5], Laurence Marrama[6] and Dominique J. Bicout[7,8]

[1]University of Twente, Faculty of Geo-Information Science and Earth Observation, PO Box 217, 7500 AE, Enschede, the Netherlands

[2]EID Mediterranee, 165 Avenue Paul Rimbaud, 34184 Montpellier, France

[3]Centro Agricoltura Ambiente "G. Nicoli", Via Argini Nord 3351, 40014 Crevalcore, Italy

[4]USDA-ARS, European Biological Control Laboratory, Tsimiski 43, 54623 Thessaloniki, Greece

[5]University of Novi Sad, Faculty of Agriculture, Laboratory for Medical Entomology, Trg D. Obradovica 8, 21000 Novi Sad, Serbia

[6]ECDC, European Centre for Disease Prevention and Control, Tomtebodavagen 11A, 17183 Stockholm, Sweden

[7]Biomathematics and Epidemiology EPSP-TIMC, VetAgro Sup, Veterinary Campus of Lyon, F – 69280 Marcy l'Etoile, France

[8]Laue – Langevin Institute, Theory Group, F – 38042 Grenoble cedex 9, France

Correspondence: bicout@ill.fr; dominique.bicout@vetagro-sup.fr

Table S1: AIC scores for the different models

	Linear model with Temperature	(S)ARIMA	(S)ARIMA + Temperature	(S)ARIMA + Precipitation	(S)ARIMA + Relative Humidity	(S)ARIMA + Temperature and Precipitation	(S)ARIMA + Temperature and Relative humidity	(S)ARIMA + Precipitation and Relative humidity	(S)ARIMA + Temperature, Precipitation and Relative humidity
Camargue	-299.4	-378.6	-369.7	-373.8	NA	367.7	NA	NA	NA
Evros	-137.3	-178.6	-178.7	-172.1	-166.0	-180.4	-178.7	-164.1	-176.7
Thessaloniki	-474.9	-670.5	-660.3	-638.5	-655.6	-661.4	-255.2	-640.2	-648.3
Emilia Romagna	-851.1	-1182.2	-1125.8	-1122.7	-1126.8	-1123.8	-1128.5	-1124.8	-1126.5
Po Delta	-1319.8	-1838.3	-1792.4	-1786.8	1786.7	-1790.7	-1790.5	-1785.2	-1788.8
Serbia	-1203.7	-1631.7	-1574.5	-1577.9	-1576.2	-1577.4	-1576.3	-1578.1	-1579.8

Table S2: MAE scores for the different models

	Linear model with Temperature	(S)ARIMA	(S)ARIMA + Temperature	(S)ARIMA + Precipitation	(S)ARIMA + Relative Humidity	(S)ARIMA + Temperature and Precipitation	(S)ARIMA + Temperature and Relative humidity	(S)ARIMA + Precipitation and Relative humidity	(S)ARIMA + Temperature, Precipitation and Relative humidity
Camargue	0.076	0.086	0.086	0.086	NA	0.086	NA	NA	NA
Evros	0.068	0.086	0.088	0.086	0.091	0.087	0.088	0.091	0.088
Thessaloniki	0.048	0.050	0.049	0.050	0.048	0.050	0.053	0.050	0.052
Emilia Romagna	0.037	0.038	0.035	0.034	0.034	0.035	0.035	0.034	0.035
Po Delta	0.041	0.039	0.035	0.035	0.035	0.035	0.035	0.035	0.035
Serbia	0.038	0.029	0.029	0.028	0.029	0.029	0.029	0.028	0.029

Figure S1: correlations between weather variables per functional unit (FU)

France: Camargue

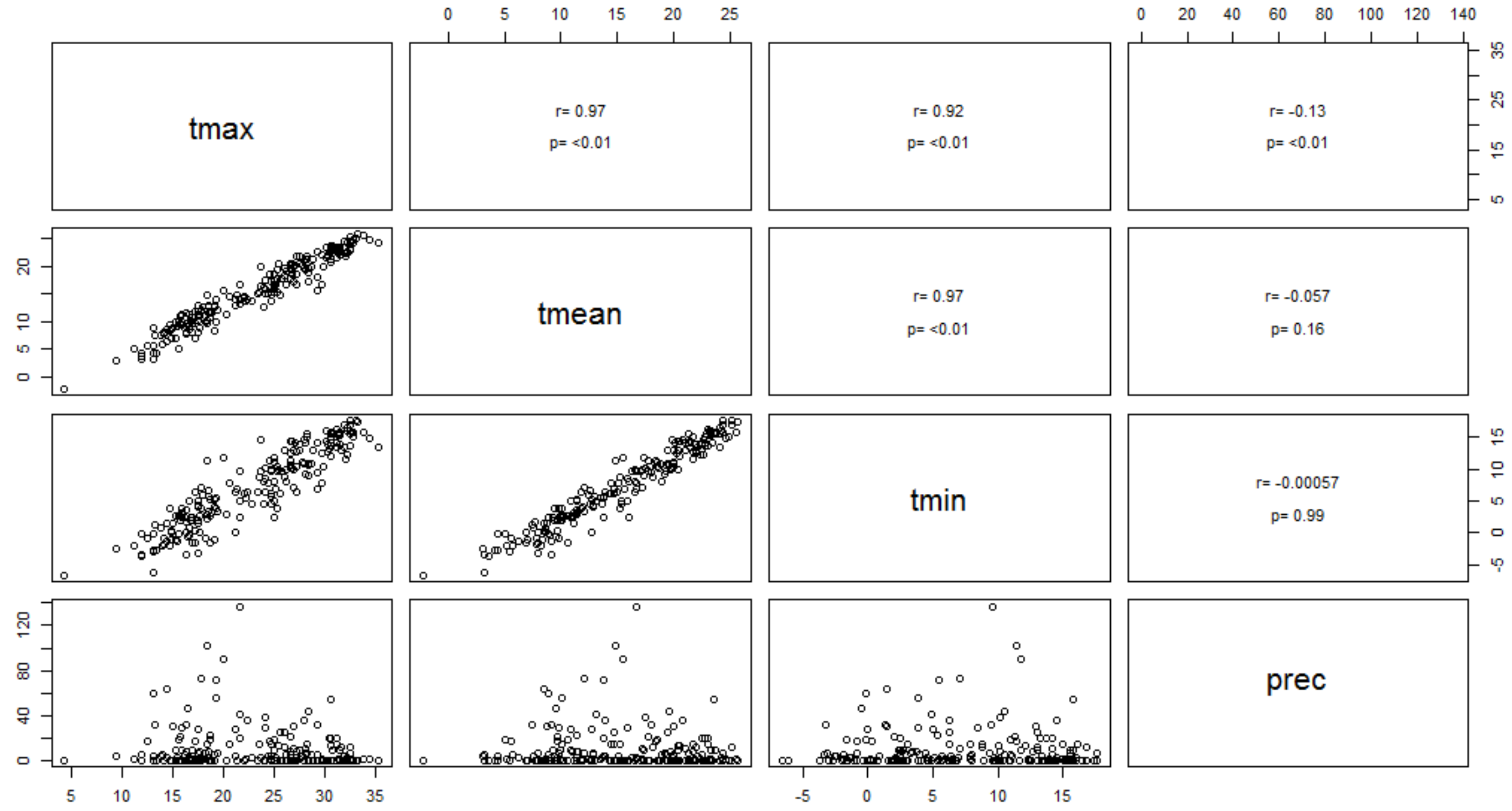

Greece: Evros

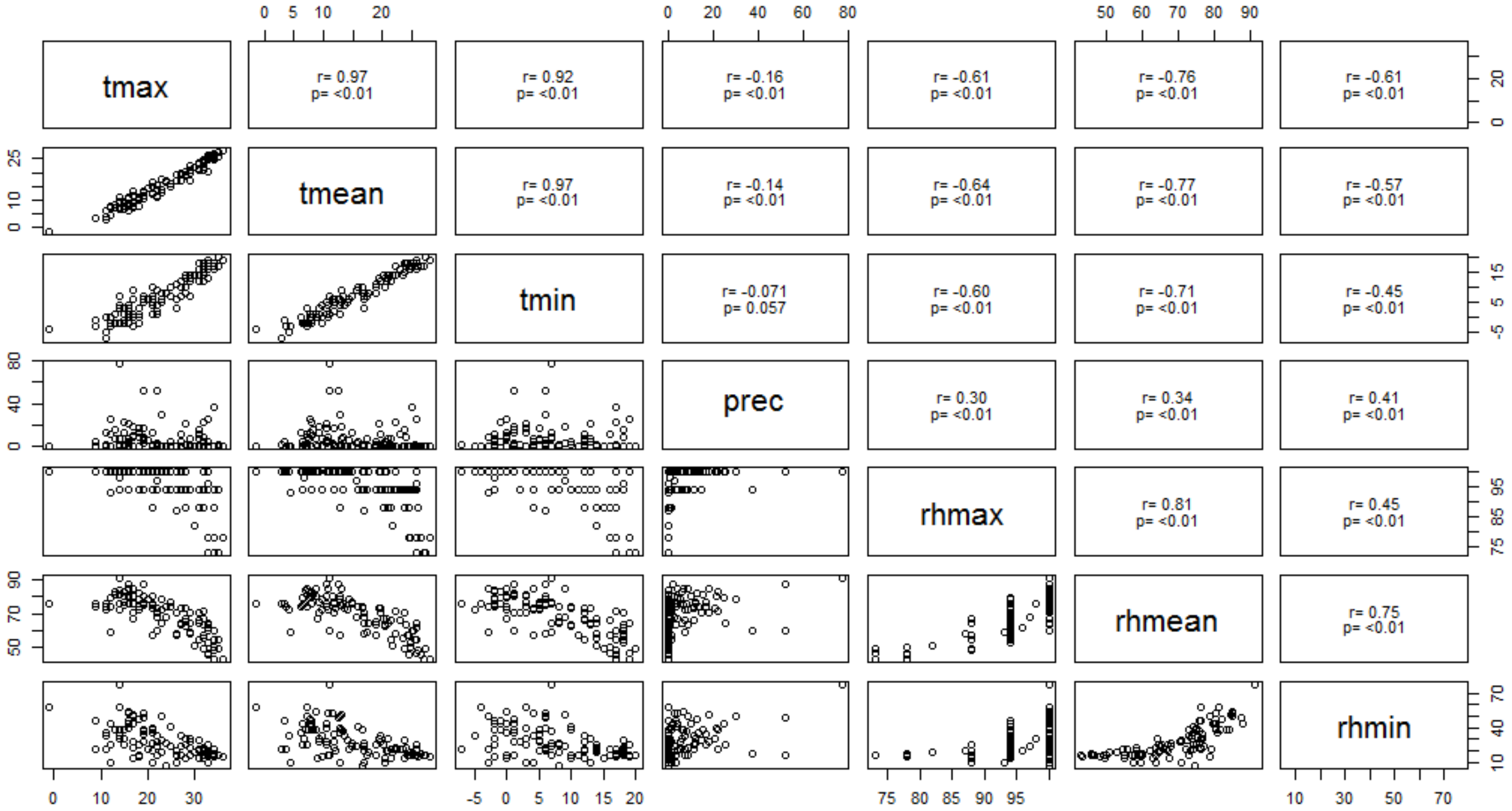

Greece: Thessaloniki

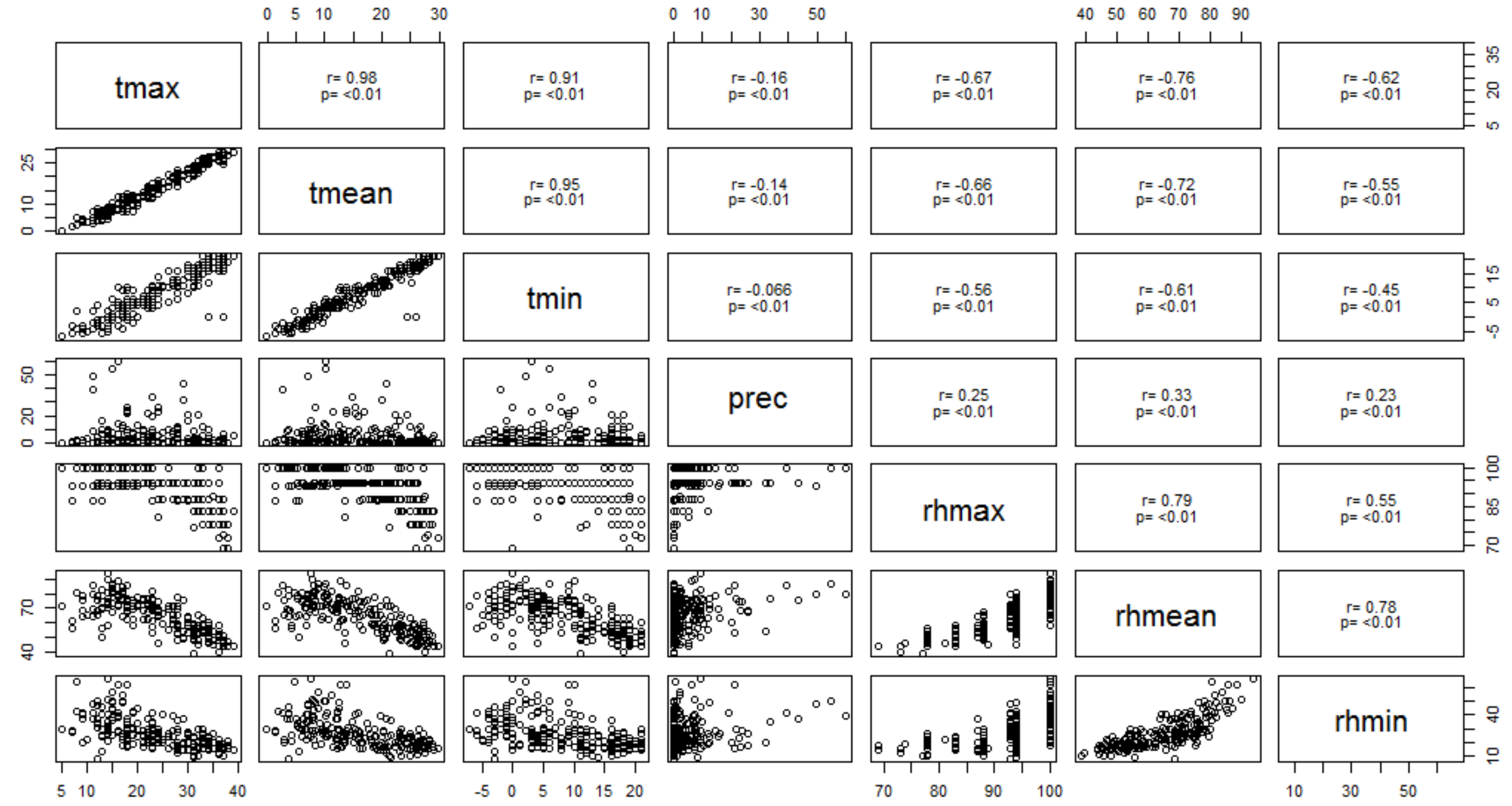

Italy: Emilia Romagna

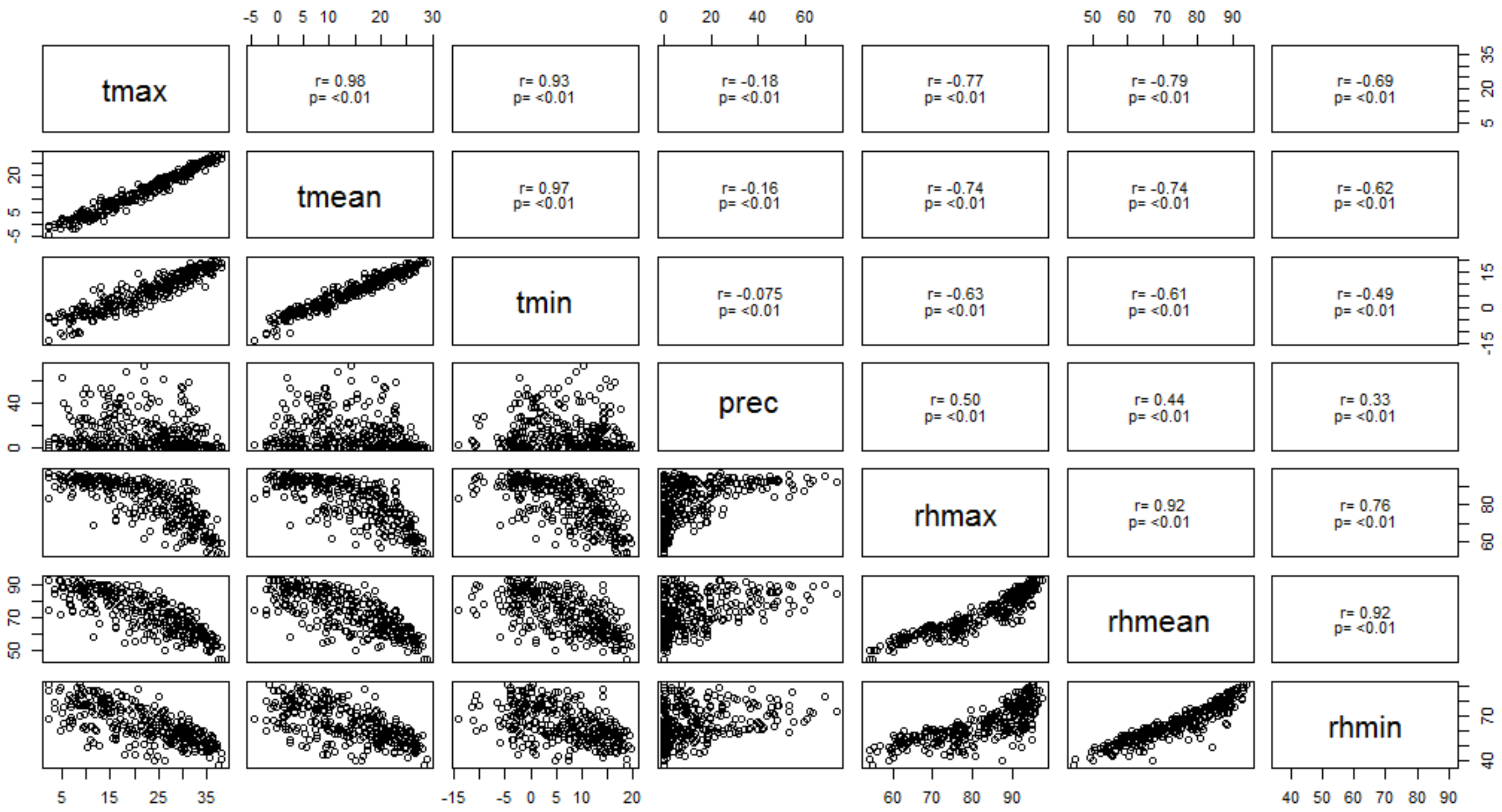

Italy: Po Delta

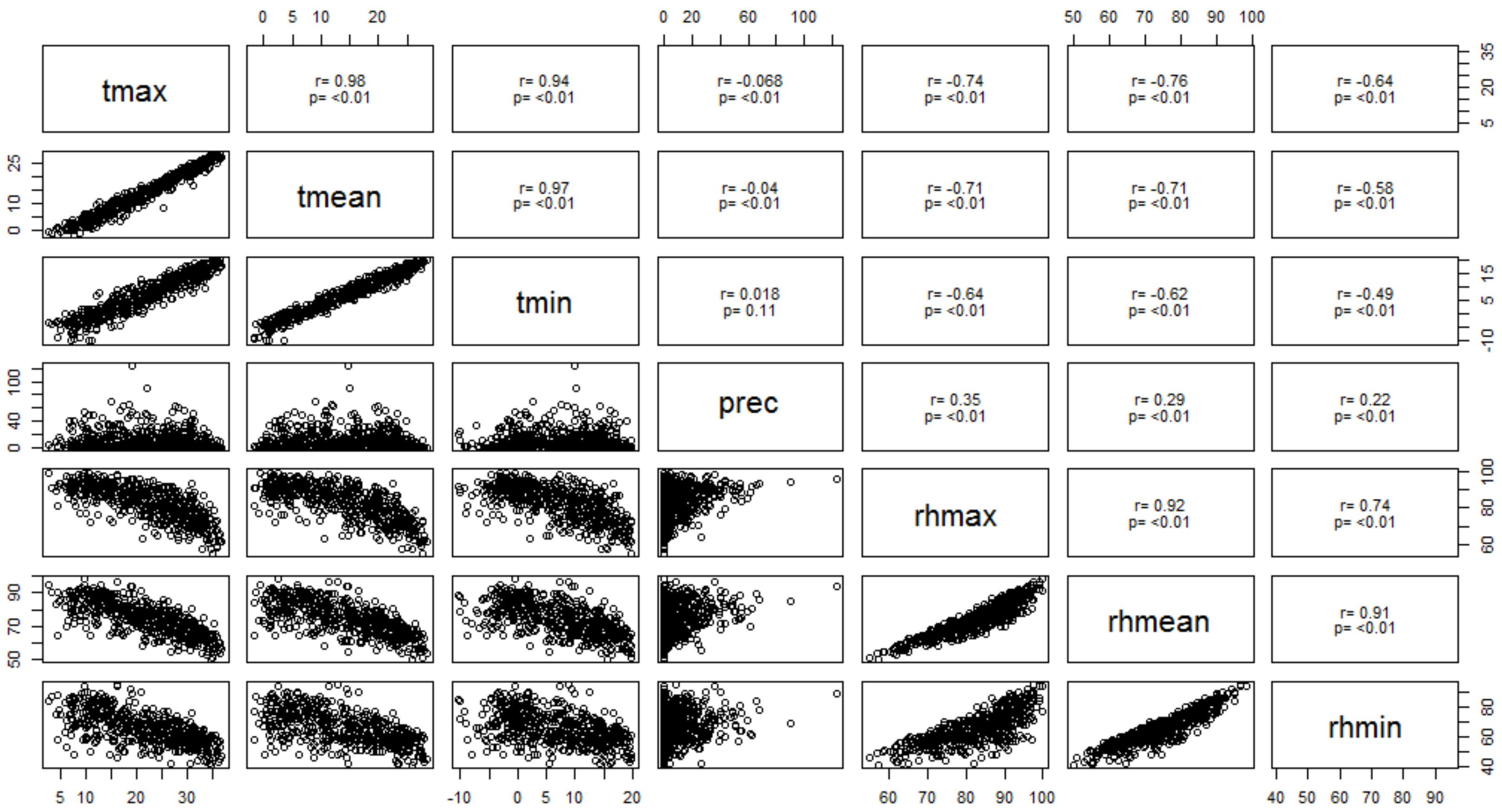

Serbia: Novi Sad and surroundings

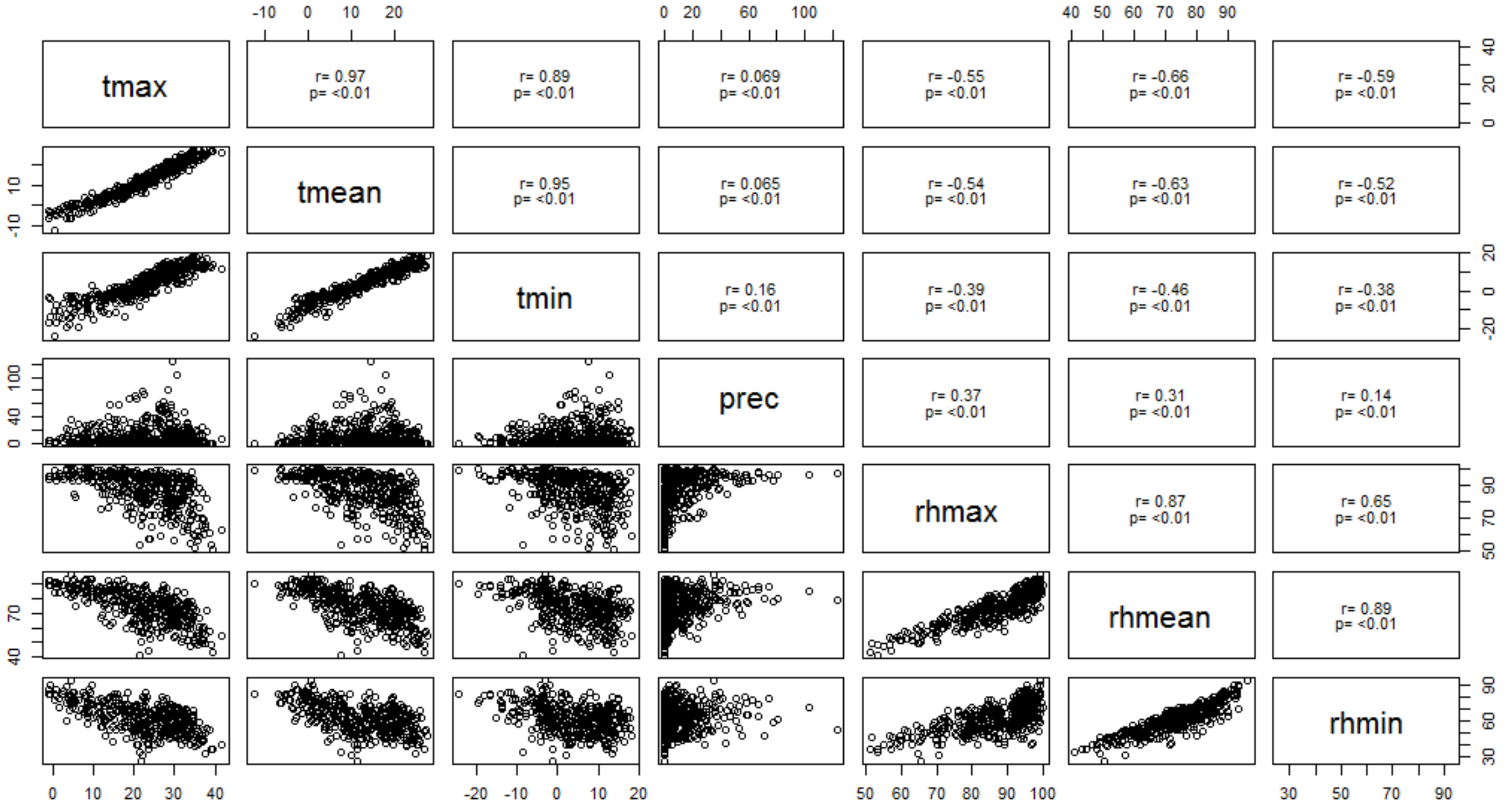

Supplement: Additional file 1: — AIC (Table S1) and MAE (Table S2) scores for the different models and correlations between weather variables per functional unit (Fig. S1). (PDF 478 kb) [file 13071_2017_2484_MOESM1_ESM.pdf]
